# Identification of COPA as a potential prognostic biomarker and pharmacological intervention target of cervical cancer by quantitative proteomics and experimental verification

**DOI:** 10.1186/s12967-021-03218-1

**Published:** 2022-01-06

**Authors:** Huiqiong Bao, Xiaobin Li, Zhixing Cao, Zhihong Huang, Li Chen, Mingbing Wang, Jiali Hu, Wenting Li, Hongwei Sun, Xue Jiang, Ping Mei, Huawen Li, Ligong Lu, Meixiao Zhan

**Affiliations:** 1 The Second School of Clinical Medicine, Southern Medical University, Department of Gynaecology, Guangzhou, China; 2 Zhuhai Precision Medical Center, Zhuhai People’s Hospital (Zhuhai Hospital Affiliated With Jinan University), Zhuhai, China; 3 Department of Pathology, Zhuhai People’s Hospital (Zhuhai Hospital Affiliated With Jinan University), Zhuhai, China; 4 Zhuhai Center for Maternal and Child Health Care, Zhuhai Women and Childen’s Hospital, Zhuhai, China; 5 Department of Gynecology, Zhuhai People’s Hospital (Zhuhai Hospital Affiliated With Jinan University), Zhuhai, China; 6 Department of Gynaecology, Guangdong Provincial People’s Hospital, Guangdong Academy of Medical Sciences, Guangzhou, China; 7 Center of Intervention Radiology, Zhuhai Precision Medicine Center, Zhuhai People’s Hospital (Zhuhai Hospital Affiliated with Jinan University), Zhuhai, China

**Keywords:** Cervical cancer, Coatomer protein subunit alpha, Immunohistochemistry, Proteomics, Tumor mechanism

## Abstract

**Background:**

Cervical cancer is the most fatal gynecological carcinoma in the world. It is urgent to explore novel prognostic biomarkers and intervention targets for cervical cancer.

**Methods:**

Through integrated quantitative proteomic strategy**,** we investigated the protein expression profiles of cervical cancer; 28 fresh frozen tissue samples (11 adenocarcinoma (AC), 12 squamous cell carcinoma (SCC) and 5 normal cervixes (HC)) were included in discover cohort; 45 fresh frozen tissue samples (19 AC, 18 SCC and 8 HC) were included in verification cohort; 140 paraffin-embedded tissues samples of cervical cancer (85 AC and 55 SCC) were used for immunohistochemical evaluation (IHC) of coatomer protein subunit alpha (COPA) as a prognostic biomarker for cervical cancer; how deficiency of COPA affects cell viability and tumorigenic ability of cervical cancer cells (SiHa cells and HeLa cells) were evaluated by cell counting kit-8 and clone formation in vitro.

**Results:**

We identified COPA is a potential prognostic biomarker for cervical cancer in quantitative proteomics analysis. By retrospective IHC analysis, we additionally verified the proteomics results and demonstrated moderate or strong IHC staining for COPA is an unfavourable independent prognostic factor for cervical cancer. We also identified COPA is a potential pharmacological intervention target of cervical cancer by a series of in vitro experiments.

**Conclusion:**

This study is the first to demonstrate that COPA may contribute to progression of cervical cancer. It can serve as a potential prognostic biomarker and promising intervention target for cervical cancer.

**Supplementary Information:**

The online version contains supplementary material available at 10.1186/s12967-021-03218-1.

## Introduction

Cervical cancer is the most fatal gynecological carcinoma worldwide [1]. Approximately 13 million women have been threatened by this malignant tumor. Early diagnosis and accurate prediction of prognosis are essential for guiding the clinical treatment [2]. ThinPrep cytology test (TCT) [3] and high-risk human papillomavirus (HPV) DNA test [4] were used for early screening [5]. Astonishingly, however, incidence rate of cervical cancer is rising steadily [6–8]. Unfortunately, there has been no significant improvement in treatment strategies for cervical cancer in the last decades. This brings forward the urgent need for further exploring prognostic biomarkers and innovative therapies to improve the prognosis of patients with cervical cancer.

As cancer treatment continues to move toward a focus on precision medicine, omics diagnostic methods are advantageous in early diagnosing and monitoring the progression of the disease. Exploration of biological characterization will facilitate the choice of targeted therapies for cervical cancer. Proteomics has emerged as a powerful tool in identifying potential prognostic biomarkers and pharmacological intervention targets in cancers [9]. Compared to other technologies, Mass spectrometry (MS) has been widely explored in surveillance and precision treatment of various cancer, standing out for its high accuracy, sensitivity, resolution, and throughput. Technological advances have optimized the performance of MS in proteomics and other analysis [10, 11], which opens new possibilities to strengthen surveillance of cancer patients in clinical situations. However, there are limited data regarding cervical cancer [12–15].

The present study was designed to investigate global protein expression profiles of cervical cancer. We employed an integrated analysis strategy comprising MS-based quantitative proteomics and parallel reaction monitoring (PRM)-based targeted proteomics in fresh frozen tissue samples of cervical cancer and healthy cervix samples. In this study, we focused on coatomer protein subunit alpha COPA, a tumor-promoting gene. COPA is involved in the movement of vesicles within the Golgi and retrograde transport of cargo proteins between endoplasmic reticulum and Golgi [16]. Recently, COPA was reported involves in a novel carcinogenesis of hepatocarcinoma, a virus-related tumor [15, 17]. However, the function of COPA in cervical cancer is unknown. In untargeted (“discovery”) proteomics and targeted (“verification”) proteomics, COPA was consistently identified as a potential prognostic biomarker of cervical cancer. We additionally verified the expression of COPA in a large number of clinical samples of cervical cancer using IHC analysis. Furthermore, we demonstrated moderate (score 2) or strong (score 3) IHC staining for COPA was a unfavorable independent risk factor for cervical cancer. Finally, through a series of in vitro experiments, the notable contribution of COPA to the progression of cervical cancer was verified in two cervical cancer cell lines.

Together, we are the first to report COPA can serve as a potential prognostic biomarker and pharmacological intervention target for cervical cancer.

## Materials and methods

### Patients and samples

**Fig. 1:**
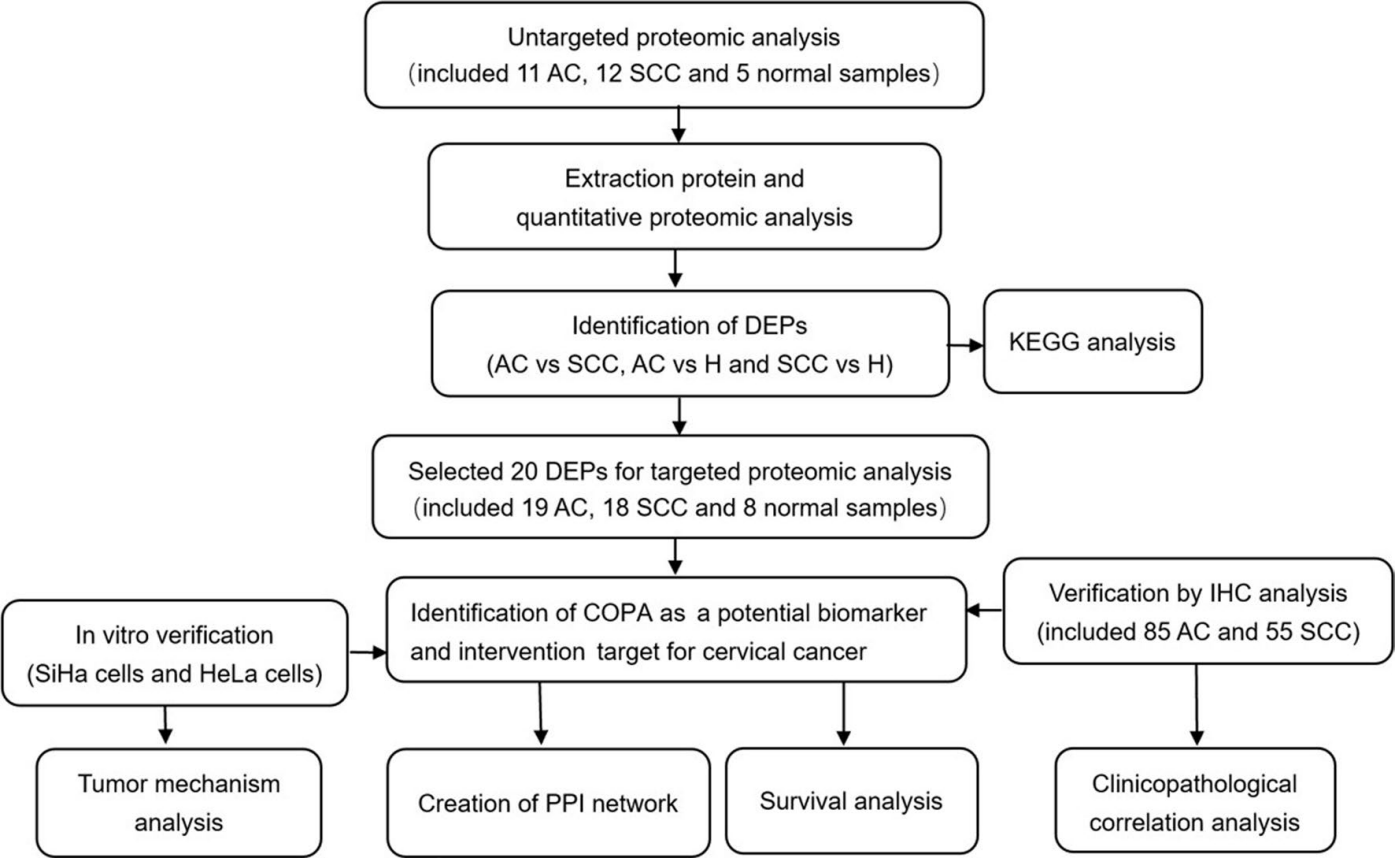
Methodological workflow of the present study

Our study was designed and analyzed according to the flow chart as shown in Fig. 1. This study has been approved by the Biomedical Ethics Committee of Guangdong Provincial People's Hospital (Permission Number: LW- [2021] No.1, KY-Q-2021–077-01) and Zhuhai People's Hospital. All of the patients provided written informed consent for their data to be used for this research. Tissue samples of from patients with cervical cancer were collected from patients who undergone radical hysterectomy and pelvic lymphadenectomy between January 2021 and May 2021 at the Department of Gynecology, Guangdong Provincial People's Hospital and the Department of Gynecology, Zhuhai People's Hospital. Normal cervix samples from patients with leiomyoma undergoing total hysterectomy during the same time period were obtained and used as healthy controls. TCT and HPV DNA tests were negative in all patients in the control group. For untargeted proteomic analysis, 28 fresh frozen tissue samples, including 11 cervical AC and 12 cervical SCC, and 5 normal cervixes were collected. For targeted proteomics analysis, 45 fresh frozen tissue samples, including 19 cervical AC and 18 cervical SCC, and 8 normal cervixes were collected. For retrospective immunohistochemistry (IHC) analysis, 155 archival formalin-fixed and paraffin-embedded (FFPE) samples of cervical cancer were included. We processed the COPA staining data by removing the samples with staining failure due to tissue detachment. Finally, 140 samples were successfully scored for COPA staining. Basic characteristics of included patients with cervical cancer were summarized in Additional file 1: Table S1, which showed no significant differences (*p* > 0.05) in three cohorts.

### Extraction and tryptic digestion of protein

Tissue samples were treated by ground into powder with liquid nitrogen, and then sonicated three times on the ice in lysis buffer (1% protease inhibitor cocktail and 8 M urea). After centrifugation at 12,000×*g* for 10 min at 4 °C, the supernatant was collected, and then the total protein concentration was determined using a BCA Protein Assay Kit according to manufacturer’s instructions. For digestion, the soluble proteins were treated with dithiothreitol (5 mM) at 56 °C for 30 min, and then they were alkylated using iodoacetamide (11 mM) at room temperature for 15 min in the dark. Then the protein samples were diluted via adding 100 mM TEAB. Eventually, trypsin was added at a trypsin-to-protein mass ratio of 1:50 in the first digestion overnight, and then a trypsin-to-protein mass ratio of 1:100 in a second digestion for 4 h.

### Quantitative proteomic analysis

Candidate protein biomarkers of cervical cancer was identified via liquid chromatography-tandem mass spectrometry. The pre-processed peptides were detected using nano spray ion (NSI) source followed by tandem MS/MS in TIMS ToF Pro, which was coupled online to UPLC system. Resulting MS/MS data were then processed applying the MaxQuant version 1.6.6.0. Tandem mass spectra were searched against the UniProt human database [18], which is a large resource of protein sequences and associated detailed annotation. We specified Trypsin/P as cleavage enzyme which allowed up to two missing cleavages. Mass tolerance of precursor ions was set as 20 ppm and 5 ppm in the first and main search, respectively. For mass tolerance for fragment ions, it was set as 20 ppm. A fixed modification was specified by the carbamidomethyl on Cys, and variable modifications were specified by acetylation modification and oxidation on Met. The false discovery rate (FDR) was set as < 1%, and the minimum score was set as > 40 for modified peptides.

### Targeted proteomics analysis

Based on potential biological functions of the identified differentially expressed proteins (DEPs), 20 candidate proteins from the discovery measurements were selected for further PRM analysis. According to the previous method of protein digestion, the protein samples were extracted from 45 fresh frozen tissues. Peptides were detected by tandem MS/MS in Q ExactiveTM Plus with UPLC system. The MS data were processed by Skyline version 3.6.

For the peptide settings, the cleavage enzyme was specified by trypsin [KR/P], and the max missing cleavage was set as two. Peptide length was set as 8–25 and we set carbamidomethyl on Cys and oxidation on Met as the variable modification, with maximum variable modifications as three.

For the transition settings, we set the precursor charges as two and three, ion charges as one and two, and ion types as b, y, and p. We set the product ions from ion three to the last ion, and the ion match tolerance was 0.02 Da.

### Retrospective immunohistochemical analysis

Paraffin sections were prepared and IHC staining were performed as described previously [19]. Rabbit monoclonal antibody targeting COPA (ab181224) were obtained from Abcam. The dilution for the primary antibody was 1:100 for COPA. In order to eliminate inter-rater variability and performed blind analysis, the percentage of positive tumor cells and staining intensity of IHC staining were scored by three experienced pathologists from the Department of Pathology in Zhuhai People's Hospital and Department of Pathology in Guangdong Provincial People's Hospital. The results were scored as follows: negative (score 0), weak (score 1), moderate (score 2) and strong (score 3) staining.

### Cell culture

Through searching the Human Protein Atlas (HPA) database (http://www.proteinatlas.org/), highly expressed COPA RNA in SiHa cells and HeLa cells was determined (Additional file 1: Fig. S1). Therefore, these two cell lines were used in the subsequent experiments. SiHa cells and HeLa cells were obtained from the American Type Culture Collection (ATCC). SiHa cells were cultured in Minimum Essential Medium (Gibco) supplemented with 10% fetal bovine serum, 100 U/ML penicillin and 100 mg/L streptomycin (Invitrogen). HeLa cells were cultured in Dulbecco's Modified Eagle Medium (Gibco) supplemented with 10% fetal bovine serum, 100 U/ML penicillin and 100 mg/L streptomycin (Invitrogen).

### Transfection of small interfering RNA and evaluation biological behaviors of cervical cancer cells

Predesigned small interfering RNA (siRNA) specific for COPA (4390824) and control siRNA (4390846) were obtained from ThermoFisher Scientific. COPA siRNA or control siRNA were introduced into SiHa cells and HeLa cells using the Lipofectamine 3000 reagent (ThermoFisher Scientific) according to the manufacturer’s instructions. To confirm the decrease of COPA protein expression in SiHa cells and HeLa cells with COPA deletion, Western blotting were performed as previously described [20]. The dilutions for the primary antibodies were as follow: 1:1000 for COPA and 1:2000 for β-actin. We observed the impact of COPA on the biological behaviors of SiHa cells and HeLa cells using cell counting kit-8 and colony formation. For determination of cell viability, a cell-counting kit 8 (Dojindo Molecular Technologies, Japan) was used according to the user manual. To detect cell tumorigenicity, clone formation assay was performed as previously described [21].

### Bioinformatics and statistics

To better understand the proteomic variances, DEPs (Differentially expressed proteins) and KEGG analysis between comparison groups were performed using ANOVA test (*p* < 0.05). We investigated the relationship among the DEPs and visualized with protein–protein interaction (PPI) networks by the STRING webserver (https://string-db.org/) [22] and Cytoscape software [23]. Kaplan–Meier survival analysis based on TCGA database was executed to explore the relationship between expression level of COPA RNA and prognosis of cervical cancer patients using GEPIA2 software [24]. For IHC analysis, the correlation between the expression level of COPA and the clinicopathological characteristics was estimated using the Mann–Whitney U test for continuous variables and Fisher's exact test or χ^2^ for categorical variables. Violin plot, heatmaps and stacked column chart were applied to demonstrate the correlation. Kaplan–Meier survival analysis was used to calculate the cumulative probability of overall survival (OS) of patients in IHC cohort, log-rank tests were used to evaluate the differences. A two-step Cox regression analysis was performed using SPSS (version 21.0) to determine the potential prognostic value of COPA in clinical situations. The hazard ratios (HRs) and their 95% confidence intervals (CIs) were also estimated. In vitro experimental phase, data were presented as mean ± standard error. Statistical significances were analyzed with one-way ANOVA using SPSS. A *p*-value < 0.05 was considered to be statistically significant.

## Results

### Screening of candidate proteins using untargeted quantitative proteomic

**Fig. 2:**
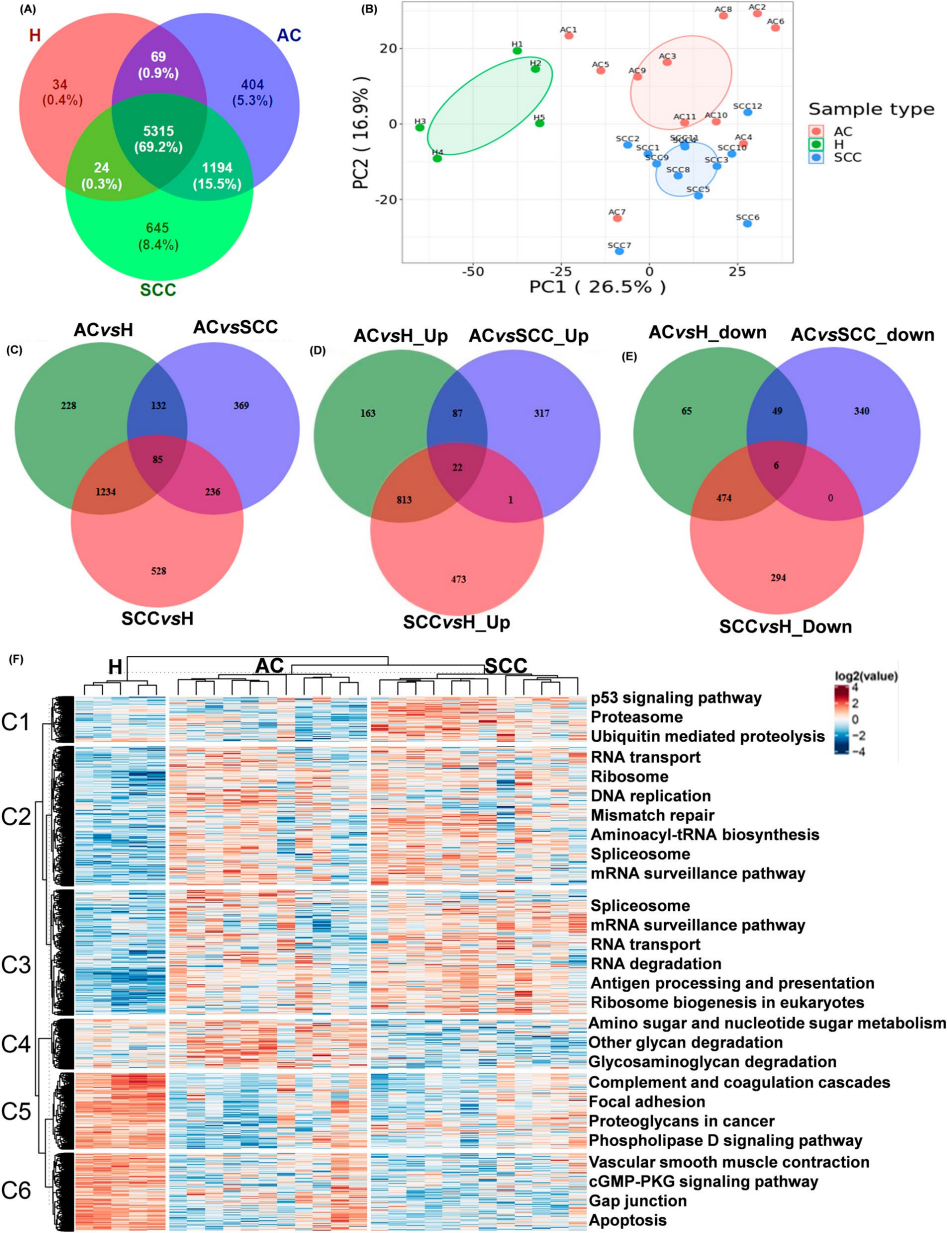
Quantitative proteomic study. **A** Venn diagram demonstrating the number of quantified proteins from the three groups. H, healthy control; AC, adenocarcinoma; SCC, squamous cell carcinoma. **B** Principal component analysis for the quantified proteins from cervical AC, cervical SCC, and healthy control groups. **C** Venn diagram of the DEPs among the three comparable groups. **D** The venn diagram showing the up-regulated DEPs among the three comparable groups. **E** Venn diagram showing the down-regulated DEPs in the three comparable groups. **F** The cluster heatmap for the significantly different proteins (*p* < 0.05) between the groups calculated by the ANOVA method, the right side of the heatmap indicating the KEGG pathway analysis

In discovery proteomics analysis, overall 7685 proteins were recognized. Among the identified proteins, 6982, 7178, and 5442 proteins were found in the cervical AC group, cervical SCC group, and healthy control group, respectively (Fig. 2A). We quantified 6602 proteins with one or more unique peptides. Compared with the healthy control group, a total of 1679 quantified proteins were significantly differentially expressed in the cervical AC group, including 1085 upregulated proteins and 594 downregulated proteins. We identified 2083 DEPs between the cervical SCC and healthy control groups, including 1309 upregulated and 774 downregulated proteins. Moreover, we identified 822 DEPs between the cervical AC and cervical SCC groups, including 427 upregulated and 395 downregulated proteins. A two-dimensional principal component analysis was executed to display the abundance variation of proteins within and among the three groups. Based on the log2-ratio of each sample over the average of all samples, we observed that cervical cancer group (cervical AC group and cervical SCC group) was completely separated from the healthy group (Fig. 2B).

We analyzed the DEPs in different comparable groups (AC vs. healthy controls, SCC vs. healthy controls, AC vs. SCC). A total of 85 DEPs overlapped across all the comparable groups. In addition, 369 DEPs were only present in the AC vs. SCC comparable group, 228 DEPs were only displayed in the AC vs. healthy controls comparable group, and 528 DEPs were only present in the SCC vs. healthy controls comparable group (Fig. 2C). The upregulated and downregulated DEPs in the three comparable groups were also analyzed. A total of 22 upregulated DEPs overlapped across all three comparable groups. In addition, 317 upregulated DEPs were only present in the AC vs. SCC comparable group, 163 upregulated DEPs were only displayed in the AC vs. healthy controls comparable group, and 473 upregulated DEPs were only present in the SCC vs. healthy controls comparable group (Fig. 2D). Six downregulated DEPs overlapped across the three comparable groups. In addition, 340 downregulated DEPs were only present in the AC vs. SCC comparable group, 65 downregulated DEPs were only displayed in the AC vs. healthy controls comparable group, and 294 downregulated DEPs were only present in the SCC vs. healthy controls comparable group (Fig. 2E).

Using KEGG database for unbiased clustering and cluster specific enrichment analysis, we found that the enriched pathways in the three groups were different (Fig. 2F). All the pathways were clustered into six clusters. Pathways enriched in the healthy group mainly included cluster5 (complement and coagulation cascades, focal adhesion, proteoglycans in cancer, and phospholipase D signaling pathway) and cluster6 (vascular smooth muscle contraction, cGMP-PKG signaling pathway, gap junction, and apoptosis). We also found that cluster4 (amino sugar and nucleotide sugar metabolism, other glycan degradation, and glycosaminoglycan degradation) was significantly enriched in the AC group, and cluster1 (p53 signaling pathway, proteasome, and ubiquitin mediated proteolysis) was specifically enriched in the SCC group.

### Validation of candidate proteins and identification COPA as a potential biomarker using targeted proteomics

**Fig. 3:**
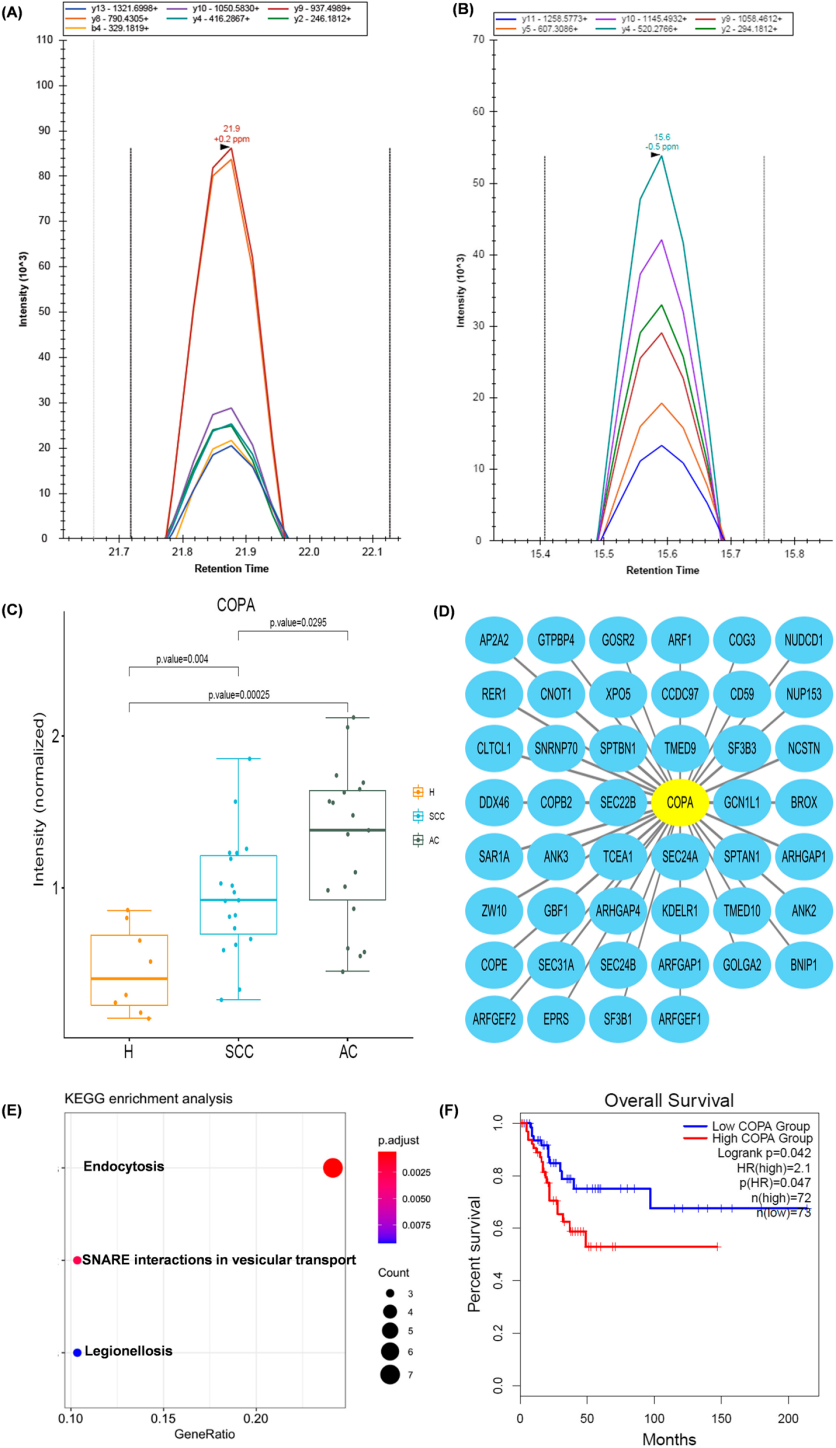
Targeted proteomics study, bioinformatics analysis of COPA. PRM verification of COPA using unique peptides: **A** GITGVDLFGTTDAVVK and **B** CPLSGACYSPEFK. **C** Box-plot exhibiting protein levels of COPA in the three comparable groups. **D** PPI network analysis of all DEPs from cervical cancer (both AC and SCC) *vs.* healthy controls showed the first neighbour relationships (undirected) to COPA. **E** KEGG pathway analysis of the COPA and its first neighbour relationships. **F** Kaplan–Meier analyses for COPA (log-rank tests) based on TCGA database using the GEPIA2 software

In targeted proteomics, 19 proteins were successfully quantified and verified. Among them, seven proteins were significantly up-regulated (*p* < 0.05) and five proteins were down-regulated (*p* < 0.05) in cervical SCC versus healthy controls (*p* < 0.05); Six proteins were significantly up-regulated (*p* < 0.05) and four proteins were down-regulated (*p* < 0.05) in cervical AC versus healthy controls; Six verified proteins were significantly up-regulated, while four verified proteins were down-regulated in cervical AC compared to cervical SCC (Table 1). Based on quantification of the following two unique peptides: GITGVDLFGTTDAVVK (Fig. 3A) and CPLSGACYSPEFK (Fig. 3B), COPA was verified as a potential biomarker of cervical cancer via targeted proteomics. COPA was observed to up-regulated significantly in cervical AC group versus healthy control group, in the cervical SCC group versus healthy control group, and in the cervical AC group versus cervical SCC group with a fold change (FC) of 2.8 (*p* = 0.000253), 2.07 (*p* = 0.004) and 1.35 (*p* = 0.0295), respectively (Fig. 3C). Then, further bioinformatics analysis and clinical value analysis of COPA were performed.

**Table 1:** PRM verified proteins in targeted proteomics analysis

Protein Accession	Gene name	SCC/H Ratio	SCC/H *p* value	AC/H Ratio	AC/H p value	AC/SCC Ratio	AC/SCC *p* value
P53621	COPA	2.07	4.00E−03	2.80	2.53E−04	1.35	2.95E−02
Q7Z3K3	POGZ	4.74	1.46E−04	6.43	4.98E−05	1.36	5.30E−02
Q8N2K0	ABHD12	2.86	2.12E−06	3.93	6.60E−03	1.37	1.11E−01
P04275	VWF	0.72	1.30E−01	0.75	2.07E−01	1.04	7.68E−01
Q6YHK3	CD109	1.08	6.71E−01	0.47	1.05E−03	0.44	3.49E−05
P52209	PGD	5.00	1.83E−03	3.00	1.12E−02	0.60	2.74E−02
P27482	CALML3	6.85	4.03E−03	0.93	9.36E−01	0.14	5.31E−05
P02671	FGA	0.35	1.99E−02	0.54	1.06E−01	1.53	2.61E−02
P02679	FGG	0.42	1.23E−02	0.61	8.61E−02	1.45	1.83E−02
P00568	AK1	0.90	5.27E−01	1.23	3.65E−01	1.37	5.33E−02
Q9UIJ7	AK3	0.96	7.95E−01	0.95	8.07E−01	0.99	9.55E−01
P43034	PAFAH1B1	1.31	1.26E−01	1.27	1.12E−01	0.97	7.92E−01
Q14624	ITIH4	0.48	7.14E−05	0.56	1.23E−03	1.16	2.92E−01
P01860	IGHG3	0.46	2.71E−05	0.44	1.32E−04	0.95	7.86E−01
Q9H223	EHD4	1.22	2.67E−01	1.33	1.35E−01	1.09	5.04E−01
P42765	ACAA2	0.97	8.73E−01	1.76	1.26E−01	1.81	1.80E−02
P02649	APOE	0.28	5.01E−06	0.46	6.79E−03	1.64	6.50E−02
Q9BSJ8	ESYT1	1.63	4.62E−04	1.62	7.65E−03	0.99	9.46E−01
O60716	CTNND1	5.21	5.00E−04	3.34	3.27E−04	0.64	1.79E−02

### Functional analysis of COPA using bioinformatics

To obtain an unbiased overview of COPA function in a biological context, the STRING webserver and Cytoscape software were used to create a PPI network including all DEPs from cervical cancer group (both AC and SCC) vs. healthy controls. A total of 45 DEPs with first neighbour relationships (undirected) to COPA were found (Fig. 3D). The analysis of the KEGG pathway shows that the 46 DEPs were mainly enriched in the following three pathways: SNARE interactions in vesicular transport, legionellosis and endocytosis pathway (Fig. 3E). Based on TCGA database, the KM curve indicated that the prognosis (quartile survival) of patients with high COPA RNA expression were significantly worse (*p* = 0.042), (Fig. 3F). The hazard ratio (HR) based on the Cox PH model was calculated, high expressed COPA RNA was an independent prognostic factor of cervical cancer with an HR of 2.1 (*p* = 0.047). Therefore, we can speculate that high expressed COPA protein may also be an unfavorable prognostic factor of patients with cervical cancer. Subsequently, the expression of COPA protein in cervical cancer tissues was additionally verified in IHC cohort.

### Correlation analysis between COPA expression and clinicopathological characteristics in IHC cohort

**Fig. 4:**
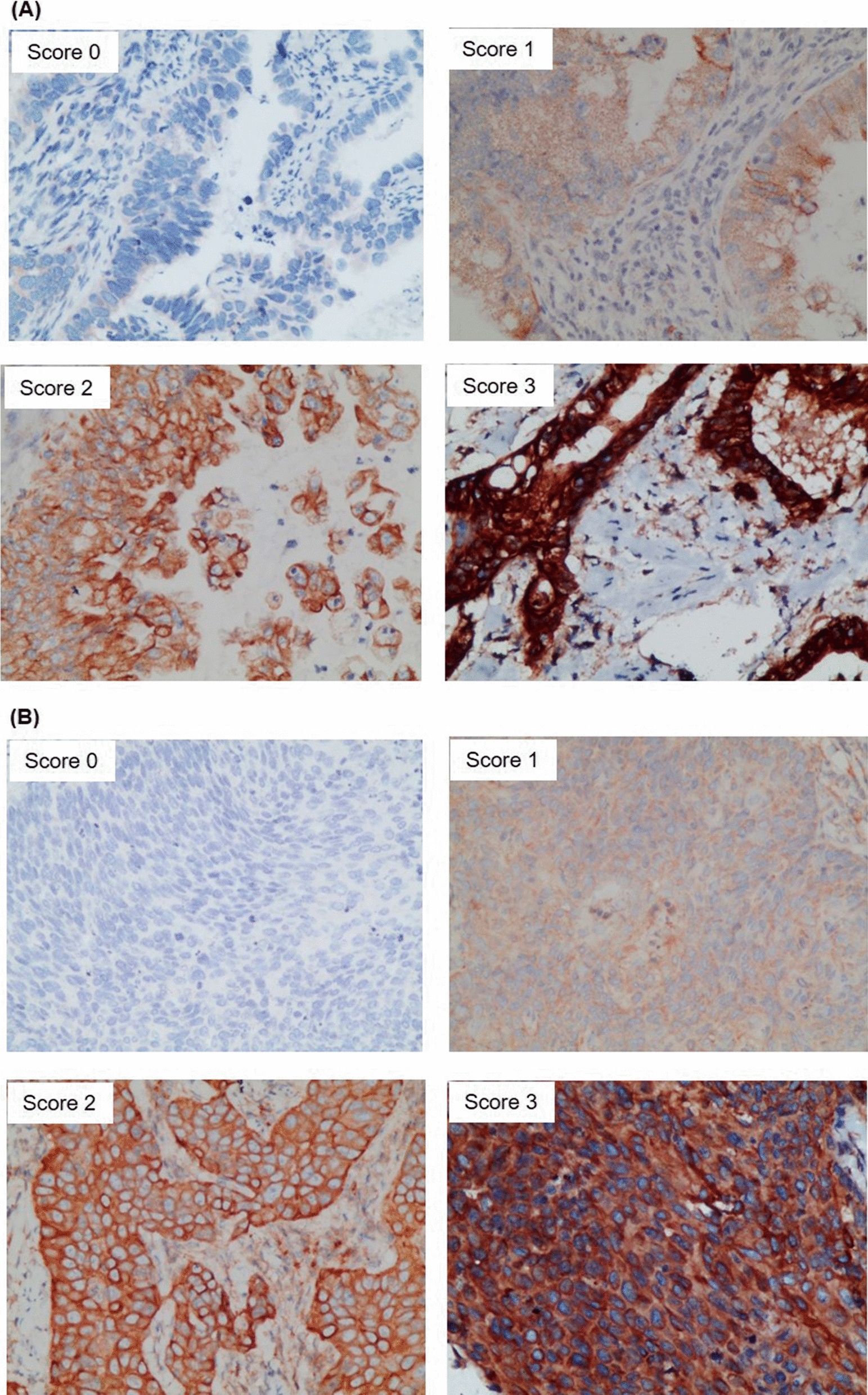
IHC staining for COPA in cervical cancer. Representative photomicrographs of IHC staining for COPA (score 0–3) in tissue samples of **A** cervical AC and **B** cervical SCC. AC, adenocarcinoma; COPA, coatomer protein subunit alpha; IHC, immunohistochemistry; SCC, squamous cell carcinoma

Human Protein Atlas analyses for the COPA (https://www.proteinatlas.org/ENSG00000122218-COPA/cell) predicted that COPA protein would be detected in the nucleoplasm, cytosol, and Golgi apparatus (Additional file 1: Fig. S2). COPA protein was retrospectively evaluated in a larger cohort of patients with cervical cancer using IHC analysis (Fig. 4), 140 patients were successfully scored for COPA staining. There were 137 (97.9%) samples were positively stained for COPA. We further investigated the correlation between COPA staining and clinicopathological characteristics of patients with cervical cancer. The clinicopathological characteristics of patients and the distribution of COPA staining were shown in Table 2. The specific results of the analysis are as follows:

**Table 2:** Correlation analysis between COPA expression and clinicopathological characteristics

Clinicopathological characteristics	Cervical AC (n = 85)				Cervical SCC (n = 55)			
	Score 0 n (%)	Score 1 n (%)	Score 2 + n (%)3	*P* value	Score 0 n (%)	Score 1 n (%)	Score 2 + n (%)3	*p* value
Stage								
I	1 (1.2)	7 (8.2)	50 (58.8)	0.455	2 (3.6)	18 (32.7)	27 (49.1)	0.536
II	0 (0)	0 (0)	20 (23.5)		0 (0)	1 (1.8)	5 (9.1)	
III	0 (0)	0 (0)	7 (8.2)		0 (0)	0 (0)	2 (3.6)	
PI								
Yes	0 (0)	0 (0)	2 (2.4)	1	0 (0)	1 (1.8)	1 (1.8)	1
No	1 (1.2)	7 (8.2)	75 (88.2)		2 (3.6)	18 (32.7)	33 (60.0)	
LNM								
Yes	0 (0)	1 (1.2)	10 (11.8)	1	1 (1.8)	2 (3.6)	6 (10.9)	0.331
No	1 (1.2)	6 (7.1)	67 (78.8)		1 (1.8)	17 (30.9)	28 (50.9)	
TS								
≥ 2 cm	1 (1.2)	1 (1.2)	46 (54.1)	0.040	2 (3.6)	14 (25.5)	24 (43.6)	1
< 2 cm	0 (0)	6 (7.1)	31 (36.5)		0 (0)	5 (9.1)	10 (18.1)	
DSI								
Yes	0 (0)	3 (3.5)	43 (50.6)	0.558	2 (3.6)	14 (25.5)	23 (41.8)	0.880
No	1 (1.2)	4 (4.7)	34 (40.0)		0 (0)	5 (9.1)	11 (20.0)	
LVSI								
Yes	0 (0)	1 (1.2)	14 (16.5)	1	1 (1.8)	6 (10.9)	16 (29.1)	0.551
No	1 (1.2)	6 (7.1)	63 (74.1)		1 (1.8)	13 (23.6)	18 (32.7)	

Stage, FIGO staging of carcinoma of the cervix uteri (2018); TS, Tumour size; DSI, Deep stromal invasion; LVSI, Lymphatic vascular space involvement; LNM, Lymph node metastasis. *p* < 0.05 is marked with asterisk

#### Histology

**Fig. 5:**
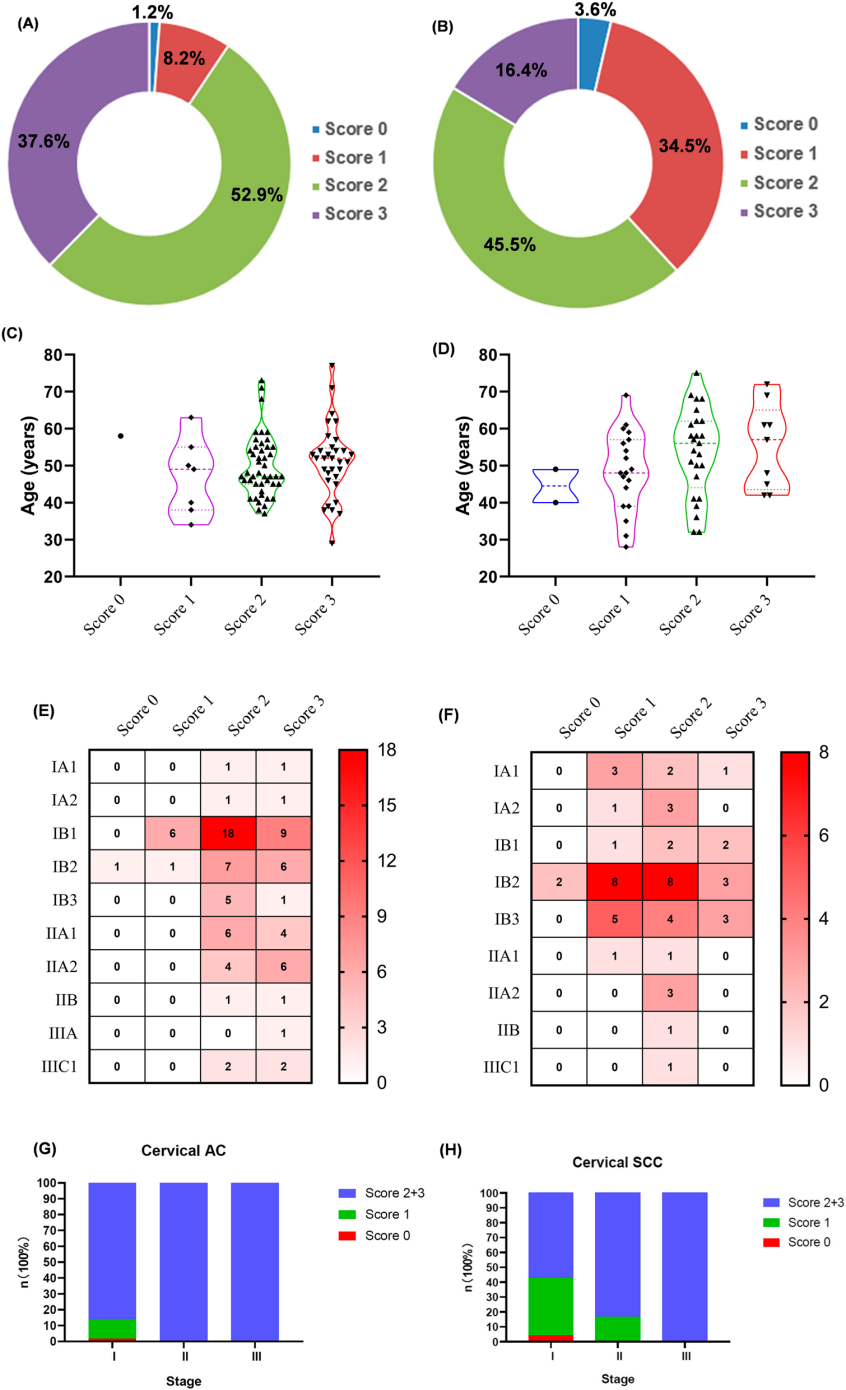
Distribution characteristics of IHC staining for COPA in cervical cancer. **A** Relative proportions of COPA staining in cervical AC scored as negative (score 0), weak (score 1), moderate (score 2), and strong (score 3) staining. **B** Relative proportions of COPA staining in cervical SCC scored as negative (score 0), weak (score 1), moderate (score 2), and strong (score 3) staining. **C** Violin plot displays the correlation of COPA staining in cervical AC (score 0, 1, 2, 3) and age. **D** Violin plot displays the correlation of COPA staining in cervical SCC (score 0, 1, 2, 3) and age. **E** Heatmap displays the distribution of COPA staining in cervical AC (score 0, 1, 2, 3) of each stage. **F** Stacked column chart shows the percentage of COPA staining in cervical AC (score 0, 1, 2, 3) of each stage. **G** Heatmap displays the distribution of COPA staining in cervical SCC (score 0, 1, 2, 3) of each stage. **H** Stacked column chart shows the percentage of COPA staining in cervical SCC (score 0, 1, 2, 3) of each stage

As shown in Fig. 5 and Table 2, the positive rate in cervical AC and cervical SCC was 98.8% (Fig. 5A) and 96.4% (Fig. 5B), respectively. Three (2.1%) sample was negative (score 0) stained for COPA in the cervical cancer sample. Only one (1.2%) tissue sample was negative (score 0) stained for COPA in the cervical AC, and 2 (3.6%) tissue sample was negative (score 0) stained for COPA in the cervical SCC. The expression of COPA in cervical AC was mainly (90.6%) moderate (score 2) and strong (score 3) expression; 8.2% of the patients with cervical AC were weakly positive for COPA. In comparison, moderate (score 2) and strong (score 3) expression of COPA in cervical SCC was less, that is 61.8%; 34.5% of the patients with cervical SCC were weakly positive for COPA.

#### Age

The frequency distribution of the age of 140 patients with cervical cancer was displayed in Additional file 1: Figure S3. The violin plot indicated the distribution of ages of patients with different expressions of COPA. The lower quartile, median, and upper quartile ages of cervical AC patients with weak (score 1) COPA expression were 38 years, 49 years, and 55 years, respectively. For the cervical AC patients with moderate (score 2) COPA expression, the median age was 47 years, while the lower and upper quartiles were 45 years and 55 years, respectively. Among the 32 cervical AC patients with strong COPA expression (score 3), the lower quartile, median, and upper quartile ages were 46.25 years, 52 years, and 54.75 years, respectively (Fig. 5C). The lower quartile, median, and upper quartile ages of cervical SCC patients with weak (score 1) COPA expression were 39 years, 48 years, and 57 years, respectively. For the cervical SCC patients with moderate (score 2) COPA expression, median age was 56 years, and lower quartile and upper quartile were 44 years and 62 years, respectively. Among the nine cervical SCC patients with strong COPA expression (score 3), the lower quartile, median, and upper quartile ages were 43.5 years, 57 years, and 65 years, respectively (Fig. 5D). We found that the median age of patients with low expression of COPA was similar in the two pathological types of cervical cancer, but the median age of patients with moderate (score 2) and strong (score 3) expression of COPA in cervical AC were relatively younger than that of in cervical SCC.

#### Stage

The FIGO stage of 140 patients ranged from stage IA1 to IIIC1. Most of patients were distributed in stages IB1 to IIA2, with stage IB1 being the most dominant, followed by stage IB2. Negative (score 0) or weak (score 1) expression of COPA in cervical AC patients were all diagnosed as stage IB, including 6 cases of stage IB1 and 2 cases of stage IB2; Even in stage I patients with cervical AC, 86.2% had moderate (score 2) and high expression of COPA. The strong expression of COPA was found in all cervical AC patients diagnosed with stage IIA and above (Fig. 5E). Similarly, cervical SCC patients with negative COPA expression (score 0) were all diagnosed as stage IB2; However, the positive expression of COPA dispersed in cervical SCC were relatively scattered than that in cervical AC; 94.7% patients of cervical SCC with weak COPA expression (score 1) were diagnosed as stage I (including 15.8% for stage IA1, 5.3% for stage IA2, 5.3% for stage IB1, 42.1% for stage IB2, and 26.3% for stage IB3) except one (5.3%) stage IIA1 patient with cervical SCC; patients with moderate COPA expression (score 2) were mainly (56.0%) categorized into stages IB (including 8.0% for stage IB1, 32.0% for stage IB2, and 16.0% for stage IB3), followed by stages IA (20.0%, including 8.0% for stage IA1, 12.0% for stage IA2) and IIA (16.0%, including 4.0% for stage IIA1, 12.0% for stage IIA2); patients of cervical SCC with high COPA expression (score 3) were mainly (88.9%) diagnosed as stage IB (including 22.2% for stage IB1, 33.3% for stage IB2, and 33.3% for stage IB3) except one (11.1%) stage IA1 patient with cervical SCC (Fig. 5F). We found that all tissues of AC (Figs. 5G) and most tissues of SCC (Figs. 5H) with stage II and above tumors were strongly positive stained of COPA (score 3), while a considerable number of tissues from patients with stage I cervical cancer were weakly stained for COPA (score 1). These results demonstrated that COPA protein level was significantly elevated in advanced cervical cancer, which suggested COPA may notable contributions to the progression of cervical cancer. High expressed COPA protein may also be an unfavorable prognostic factor of patients with cervical cancer.

#### Parametrial invasion

**Fig. 6:**
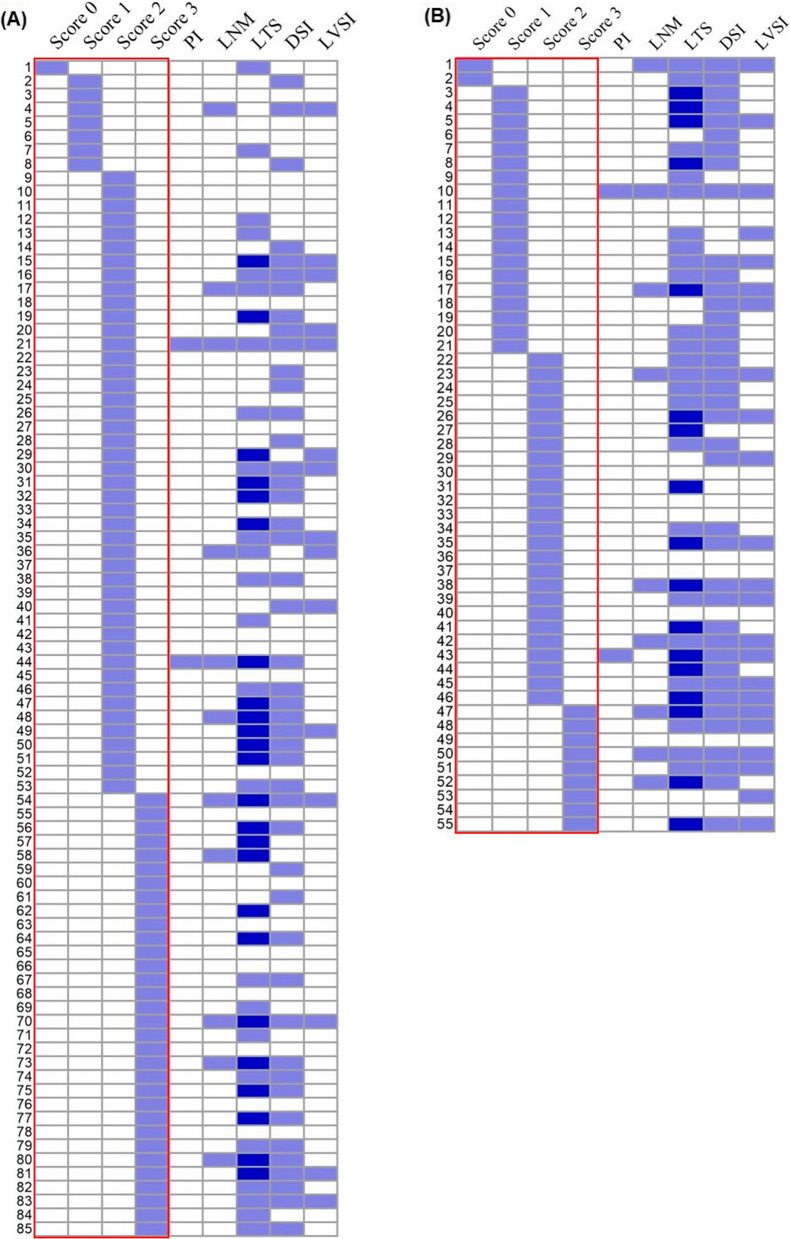
Correlation between COPA staining and histopathological risk factors of cervical cancer. **A** Correlation between COPA staining in cervical AC (score 0, 1, 2, 3) and histopathological risk factors of cervical cancer. **B** Correlation between COPA staining in cervical SCC (score 0, 1, 2, 3) and histopathological risk factors of cervical cancer

Parametrial invasion (PI) was present in four (2.9%) patients with cervical cancer, the two pathological subtypes accounted for 50% respectively; all cervical AC patients with PI were in the moderate COPA expression (score 2) group (Fig. 6A). PI was also present in two (3.6%) patients with cervical SCC, one patient with weak COPA expression (score 1) and the other with moderate COPA expression (score 2) (Fig. 6B).

#### Lymph node metastasis

Twenty patients (14.3%) of cervical cancer with lymph node metastasis (LNM) were found by histopathology, most patients (80.0%) were in the moderate COPA expression (score 2) group and the high COPA expression (score 3) group. Eleven (12.9%) patients with cervical AC were presented LNM. Of these, 90.9% patients were in the moderate COPA expression (score 2) group and the high COPA expression (score 3) group, except one (9.1%) patient with weak COPA expression (Fig. 6A). Nine (16.4%) patients with cervical SCC were found LNM. Of these, COPA was moderately (score 2) or highly (score 3) expressed in 6 (66.7%) cases, weakly expressed in 2 (22.2%) cases, and negatively expressed in one (11.1%) case (Fig. 6B).

#### Tumor size

Patients with cervical cancer were categorized into two categories as follows: tumor size < 2 cm and ≥ 2 cm: 52 (37.1%) patients were classified into the former, 88 (62.9%) patients were classified into the latter. We found tumor size of 48 (56.5%) patients with cervical AC is larger than or equal to 2 cm, moderately (score 2) or highly expressed (score 3) COPA were found in 46 (95.8%) samples. The remarkably elevated COPA was significantly correlated with tumor size ≥ 2 cm in cervical AC (*p* = 0.04). Besides, 24 cervical AC patients with bulky tumor (tumor size ≥ 4 cm) were all in the moderate (score 2) and the high (score 3) COPA expression group (Fig. 6A). Meanwhile, tumor size of 40 (72.7%) patients with cervical SCC is larger than or equal to 2 cm, moderately (score 2) or highly expressed (score 3) COPA were found in 24 (60.0%) patients; negatively (score 0) or weakly expressed (score 1) COPA were found in 16 (40.0%) cervical SCC patients (Fig. 6B).

#### Deep stromal invasion

Deep stromal invasion (DSI) was found in eighty-five (60.7%) cervical cancer samples by histopathology. Specifically, DSI was presented in 46 (54.1%) patients with cervical AC and 39 (70.9%) patients with cervical SCC; Forty-three (93.5%) cervical AC patients with DSI were in the moderate (score 2) COPA expression group or high (score 3) COPA expression group (Fig. 6A). Meanwhile, 23 (59.0%) cervical SCC patients with DSI was in the moderate (score 2) COPA expression group or high (score 3) COPA expression group (Fig. 6B).

#### Lymphatic vascular space involvement

Lymphatic vascular space involvement (LVSI) was found in 38 (27.1%) cervical cancer samples by histopathology. To be specific, LVSI was presented in 15 (17.6%) patients with cervical AC and 23 (41.8%) patients with cervical SCC; All samples (100.0%) of cervical AC with LVSI were positively expressed COPA, and 93.3% of them were in the moderate (score 2) or high (score 3) COPA expression group (Fig. 6A). In the cervical SCC samples with LVSI, 95.7% were positively expressed COPA, and 69.6% was in the moderate (score 2) or high (score 3) COPA expression group, 26.1% was in the weak (score 1) COPA expression group; negative (score 0) COPA expression was found in only one (4.3%) sample of cervical SCC with LVSI (Fig. 6B).

### Validation of COPA staining can serve as an independent prognostic factor for cervical cancer

**Fig. 7:**
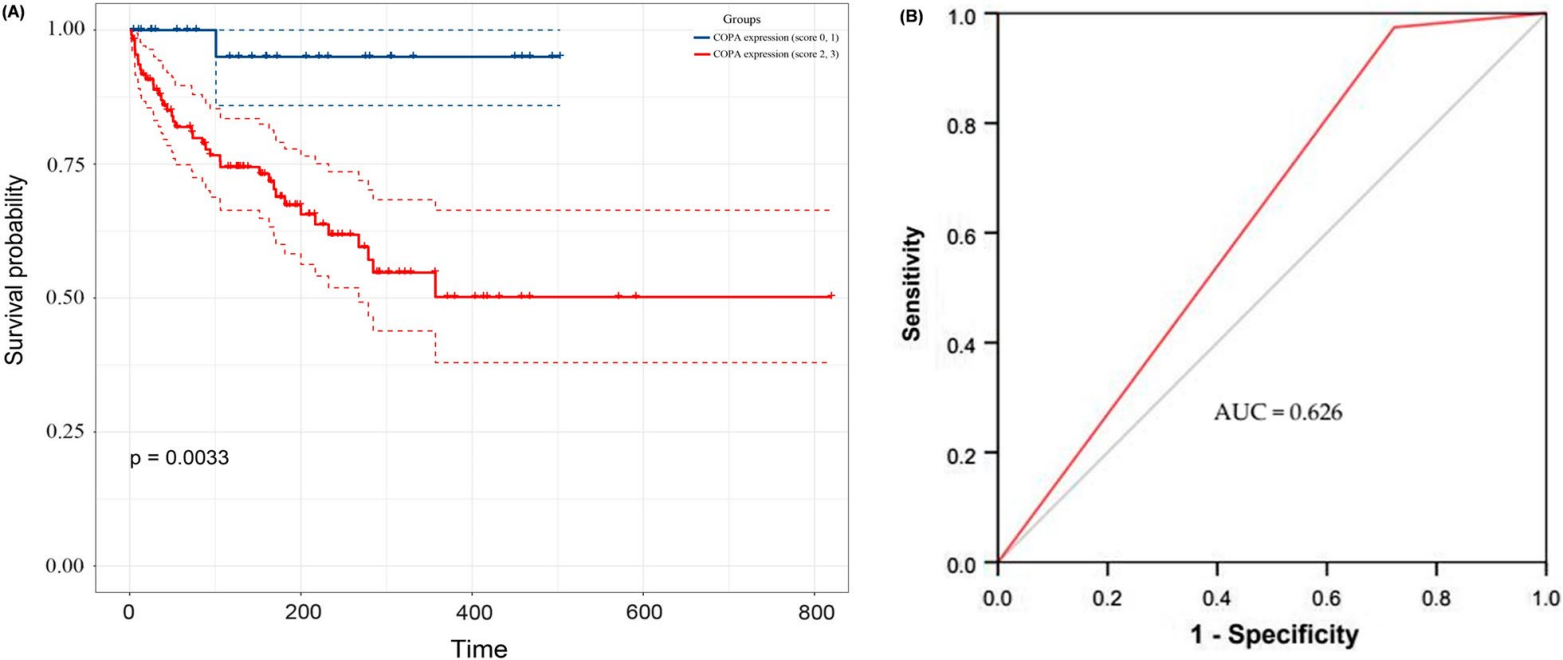
Survival analysis of COPA in clinical samples. **A** Kaplan–Meier survival analysis for cervical cancer patients with different IHC staining for COPA (score 0-1vs score 2–3). **B** ROC curve analysis for cervical cancer patients with moderate (score 2) or strong (score 3) COPA staining

Through a survival analysis, we discovered that the patients with the moderate (score 2) or strong (score 3) COPA staining possessed worse survival than the patients with the negative (score 0) or weak (score 1) COPA staining (*p* = 0.0033) (Fig. 7A). Area under the curve (AUC) of ROC curve was 0.626 (Fig. 7B). We performed a univariate Cox regression analysis to assess the influences of patients’ clinicopathological parameters and COPA staining on patients’ OS. The results indicated that moderate (score 2) or strong (score 3) staining of COPA, advanced stage and lymph node metastasis were unfavourable prognostic factors of cervical cancer. Then, we performed a multivariate Cox regression analysis, moderate (score 2) or strong (score 3) COPA staining (HR = 8.946; 95% CI: 1.218–65.714, *p* = 0.031) and advanced stage were independent prognostic factor of cervical cancer (Table 3).

**Table 3:** Univariate and multivariate Cox regression analysis

Variables	Univariate analysis			Multivariate analysis		
	HR	95% CI	*p* value	HR	95% CI	*p* value
COPA	10.773	1.479–78.497	0.019*	8.946	1.218–65.714	0.031*
Stage	5.427	2.546–11.565	< 0.001*	3.624	1.459–8.999	0.006*
LNM	2.304	1.146–4.629	0.019*	1.354	0.584–3.139	0.48
TS	1.243	0.637–2.426	0.523			
DSI	1.455	0.737–2.874	0.28			
PI	0.97	0.133–7.076	0.976			
LVSI	1.501	0.780–2.888	0.224			

Stage, FIGO staging of carcinoma of the cervix uteri (2018); TS, Tumour size; DSI, Deep stromal invasion; LVSI, Lymphatic vascular space involvement; LNM, Lymph node metastasis. *p* < 0.05 is marked with *

### Depletion COPA inhibited the aggressive behaviors of cervical cancer cells in vitro

**Fig. 8:**
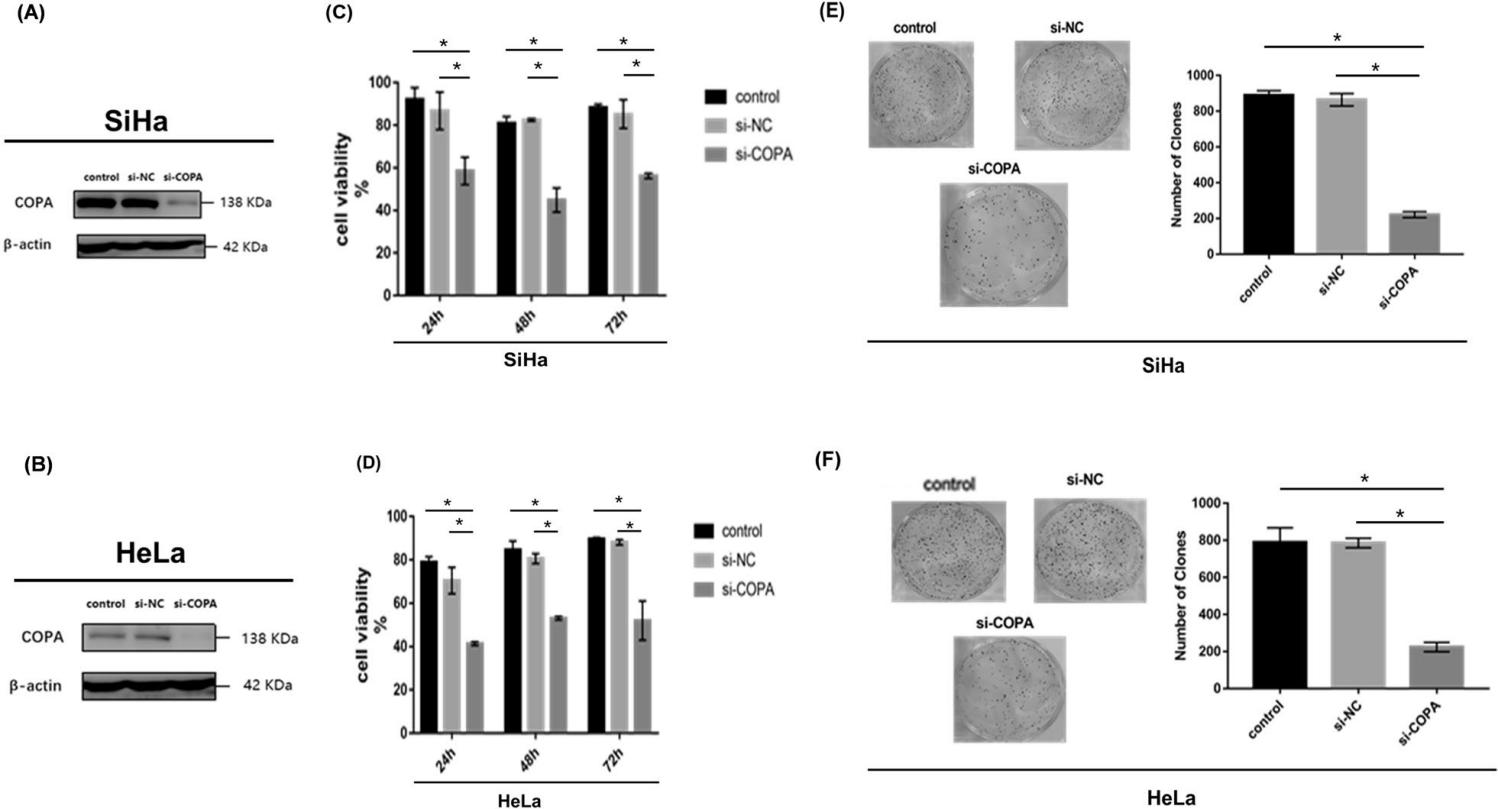
Depletion COPA inhibited the aggressive behaviors of cervical cancer cells in vitro. The effect of COPA knockdown was examined by western blotting analysis in **A** SiHa cells and **B** HeLa cells. β-actin was used as loading control. CCK8 assays showed COPA depletion inhibited cell viability of **C** SiHa cells and **D** HeLa cells. Quantification of foci formation were induced by the indicated clones of **E** SiHa cells and **F** HeLa cells. CCK8, cell counting kit-8. *p < 0.05

SiHa cells (cervical SCC) and HeLa cells (cervical AC) exhibit relatively high endogenous COPA levels. To further elucidate the functional effects of COPA in cervical cancer cells, these two cervical cancer cell lines were used in vitro intervention experiments. We observed the effects of COPA on cell viability and tumorigenic ability in the two COPA knockdown cervical cancer cells. Using Specific siRNAs for COPA, we successfully knocked out COPA in SiHa cells and HeLa cells. The protein abundances of COPA in SiHa cells (Fig. 8A) and HeLa cells (Fig. 8B) were descended robustly in response to transcriptional suppression of COPA. COPA silencing significantly inhibited cell viability in SiHa cells (*p* < 0.05) (Fig. 8C) and in HeLa cells (*p* < 0.05) (Fig. 8D). In addition, deficiency of COPA dramatically interfered the tumorigenic ability of SiHa cells (*p* < 0.05) (Fig. 8E) and  of SiHa cells (*p* < 0.05) (Fig. 8F). These observations provided solid evidence that COPA makes notable contributions to progression of cervical cancer, COPA is a novel pharmacological intervention target for cervical cancer. We demonstrated that deficiency of COPA dramatically interfered the aggressive behaviors of cervical cancer cells in vitro.

## Discussion

Protein mass spectrometry can be generally classified into untargeted (“discovery”) proteomics and targeted (“verification”) proteomics [25]. Targeted proteomics has made steady advances and was named Nature Methods’ 2012 Method of the year because of its special suitability for biomarker candidate verification and clinical applications [26]. More recent, high mass accuracy-based parallel reaction monitoring (PRM)-MS shows outstanding performance characteristics [27]. However, previous tissue-based proteomics of cervical cancer were based on untargeted proteomics. The validation data of these studies were antibody-based technologies such as western blotting and immunohistochemistry, but not targeted proteomics [15, 21, 28–37]. To our knowledge, this is the first study using the integrated analysis strategy of untargeted proteomics and targeted proteomics to investigate protein expression profiles of cervical cancer. COPA was consistently identified as a potential prognostic biomarker of cervical cancer in two stage proteomic. The validation of the proteomic findings in a large number of clinical samples is beneficial to push forward clinic applications. Therefore, additional IHC analysis of COPA in cervical cancer was performed in a large amount of FFPE samples. In IHC cohort, we not only verified the results of proteomics, but also defined the clinical value of COPA staining in cervical cancer.

Currently, surgery remains the primary treatment of cervical cancer. Adjuvant treatments (radiotherapy or chemoradiotherapy) are determined based on histopathological risk factors [38–40]. The prognosis of cervical cancer is closely related to the stage and histopathological risk factors. Accurately predict the prognosis would provide new opportunities for optimizing therapeutic protocols. Parametrical invasion, positive surgical margins, and lymph node metastasis are high-risk factors for cervical cancer. Large tumor (tumor size ≥ 4 cm), DSI, and LVSI are considered intermediate-risk factors [38–40]. Recent studies reported that cervical cancer patients with tumor size ≥ 2 cm have unfavorable 5-year overall survival [41]. Our results of IHC analysis demonstrated that COPA protein level was significantly elevated in advanced cervical cancer. Notably, the remarkably elevated COPA was significantly correlated with tumor size ≥ 2 cm in cervical AC (*p* = 0.04). The moderate (score 2) or strong COPA staining is an unfavourable independent prognostic factor of cervical cancer. These findings further demonstrates that COPA can serve as a valuable prognostic biomarker for cervical cancer and is suitable for clinical application.

COPA is part of the coatomer protein complex I, which is involved in the movement of vesicles within the Golgi and retrograde transport of cargo proteins between endoplasmic reticulum and Golgi [16]. Our functional analysis of COPA and its first neighbor DEPs found the mainly enriched pathways were SNARE interactions in vesicular transport, legionellosis and endocytosis pathway. Therefore, we speculate that COPA could serve as a tumor-promoting gene in cervical cancer trough participate in the regulation of the aforementioned pathways. Recent studies have underscored the role of COPA in a novel carcinogenic mechanism. Downregulation of intracellular COPA levels has been shown to dramatically reduce tumorigenic ability of hepatocarcinoma cells [17] and inhibit the proliferation of prostate cancer cells [42]. Since, our previous analysis showed that the level of COPA protein was significantly increased in advanced cervical cancer, we also speculated that COPA could be an attractive pharmacological intervention target of cervical cancer. One would query what is the evidence to support this speculation? To this end, we explored COPA functional role in two cervical cancer cell lines. Upon the COPA knockdown, cell viability and tumorigenesis ability of SiHa cells and HeLa cells were dramatically reduced. Therefore, COPA is critical in the progression of cervical cancer. Depletion COPA inhibited the aggressive behaviors of cervical cancer cells in vitro. So far, we have identified COPA as a potential prognostic biomarker and a pharmacological intervention target for cervical cancer on multiple levels.

Indubitably, although the results are promising, some limitations must be clarified in this study. First, we retrospectively analysed the correlation between the IHC staining of COPA and clinicopathological characteristics of patients with cervical cancer, its clinical utility would be more convincing using prospective data to assess. Second, the signalling mechanisms of COPA in cervical cancer is interpreted based on bioinformatics analyses, further experimental studies to validate our findings are imperative. Third, although we confirmed in vitro that COPA is an attractive pharmacological intervention target for cervical cancer, in vivo intervention experiments are also warranted in the future.

## Conclusions

To summarize, we analyzed protein expression profiles in cervical cancer through integrating proteomic strategy. COPA was identified as a potential prognostic biomarker for cervical cancer, which was additionally verified by IHC staining. Notable, the critical functional role of COPA in cervical cancer was determined via in vitro experiments. All our data consistently suggested that COPA can serve as a potential prognostic biomarker and pharmacological intervention target for cervical cancer. We hope that these findings will offer some useful insights for subsequent studies and clinical practice.

## Supplementary Information

**Additional file 1: Table S1**. Baseline characteristics of included patients with cervical cancer. **Table S2**. The histological types of included cervical adenocarcinoma. **Figure S1**. RNA expression of COPA in cell lines based on HPA database. (A) The cell lines ordered by human tissues and organ; (B) The cell lines ordered by descending RNA expression order. **Figure S2**. HPA (The Human Protein Atlas) analyses for the COPA predicted that the protein was detected in nucleoplasm, cytosol and Golgi apparatus and was predicted to be secreted. **Figure S3**. Histograms showing the distributions of ages of the 140 patients with cervical cancer in the IHC analysis. **Figure S4**. The positions of COPA plotted in the volcano plots of cervical cancer (AC and SCC) vs. healthy controls.

## Data Availability

All data that support the findings of this study are available from the corresponding author upon reasonable request.
