# Supplementary material for: Identification of COPA as a potential prognostic biomarker and pharmacological intervention target of cervical cancer by quantitative proteomics and experimental verification
Source: J Transl Med. 2022 Jan 6;20:18. doi: 10.1186/s12967-021-03218-1 (PMC8740354; doi:10.1186/s12967-021-03218-1)
Supplement: Supplementary file 1 — Additional file 1: Table S1. Baseline characteristics of included patients with cervical cancer. Table S2. The histological types of included cervical adenocarcinoma. Figure S1. RNA expression of COPA in cell lines based on HPA database. (A) The cell lines ordered by human tissues and organ; (B) The cell lines ordered by descending RNA expression order. Figure S2. HPA (The Human Protein Atlas) analyses for the COPA predicted that the protein was detected in nucleoplasm, cytosol and Golgi apparatus and was predicted to be secreted. Figure S3. Histograms showing the distributions of ages of the 140 patients with cervical cancer in the IHC analysis. Figure S4. The positions of COPA plotted in the volcano plots of cervical cancer (AC and SCC) vs. healthy controls. [file 12967_2021_3218_MOESM1_ESM.docx]

**Identification of COPA as a potential prognostic biomarker and pharmacological intervention target of cervical cancer by** **quantitative proteomics and experimental verification**

**Table legends:**

Table S1. Baseline characteristics of included patients with cervical cancer.

Table S2. The histological types of included cervical adenocarcinoma.

**Figure legends:**

Figure S1. RNA expression of COPA in cell lines based on HPA database. (A) The cell lines ordered by human tissues and organ; (B) The cell lines ordered by descending RNA expression order.

Figure S2. HPA (The Human Protein Atlas) analyses for the COPA predicted that the protein was detected in nucleoplasm, cytosol and Golgi apparatus and was predicted to be secreted.

Figure S3. Histograms showing the distributions of ages of the 140 patients with cervical cancer in the IHC analysis.

Figure S4. The positions of COPA plotted in the volcano plots of cervical cancer (AC and SCC) vs. healthy controls.

**Table S1. Baseline characteristics of included patients**

|  |  |  |  |  |  |  |  |  |
| --- | --- | --- | --- | --- | --- | --- | --- | --- |
| Clinicopathological characteristics |  | Discovery cohort |  | Validation cohort |  | IHC cohort |  | *p* value |
|  |  | n |  | n |  | n |  |  |
| Histology |  |  |  |  |  |  |  |  |
| SCC |  | 12 |  | 18 |  | 55 |  | 0.270 |
| AC |  | 11 |  | 19 |  | 85 |  |  |
| FIGO staging |  |  |  |  |  |  |  |  |
| I |  | 13 |  | 24 |  | 106 |  | 0.092 |
| II |  | 7 |  | 9 |  | 25 |  |  |
| III |  | 2 |  | 3 |  | 9 |  |  |
| IV |  | 1 |  | 1 |  | 0 |  |  |
| PI |  |  |  |  |  |  |  |  |
| Yes |  | 0 |  | 0 |  | 4 |  | 0.512 |
| No |  | 23 |  | 37 |  | 136 |  |  |
| LNM |  |  |  |  |  |  |  |  |
| Yes |  | 1 |  | 3 |  | 20 |  | 0.288 |
| No |  | 22 |  | 34 |  | 120 |  |  |
| Tumor size |  |  |  |  |  |  |  |  |
| <2cm |  | 5 |  | 8 |  | 52 |  | 0.102 |
| ≥2cm |  | 18 |  | 29 |  | 88 |  |  |
| DSI |  |  |  |  |  |  |  |  |
| ≤1/2 |  | 5 |  | 9 |  | 55 |  | 0.093 |
| >1/2 |  | 18 |  | 28 |  | 85 |  |  |
| LVSI |  |  |  |  |  |  |  |  |
| Yes |  | 4 |  | 8 |  | 38 |  | 0.529 |
| No |  | 19 |  | 29 |  | 102 |  |  |
| Total |  | 23 |  | 37 |  | 140 |  |  |
| Abbreviations: Stage, FIGO staging of carcinoma of the cervix uteri (2018); TS, Tumour size; DSI, Deep stromal invasion; LVSI, Lymphatic vascular space involvement; LNM, Lymph node metastasis. *p* < 0.05 is marked with asterisk (*) | | | | | | | | |

**Table S2 The histological types of cervical adenocarcinoma**

|  |  |  |  |
| --- | --- | --- | --- |
| Pathological type | Discovery cohort | Validation cohort | IHC cohort |
| Squamous cell carcinoma | 12 | 18 | 55 |
| Adenocarcinoma |  | | |
| Usual-type endocervical carcinoma | 9 | 16 | 70 |
| Villoglandular papillary carcinoma | 0 | 0 | 3 |
| Endometrioid carcinoma | 0 | 0 | 1 |
| Mucinous carcinoma | 2 | 3 | 11 |
|  |  |  |  |

**
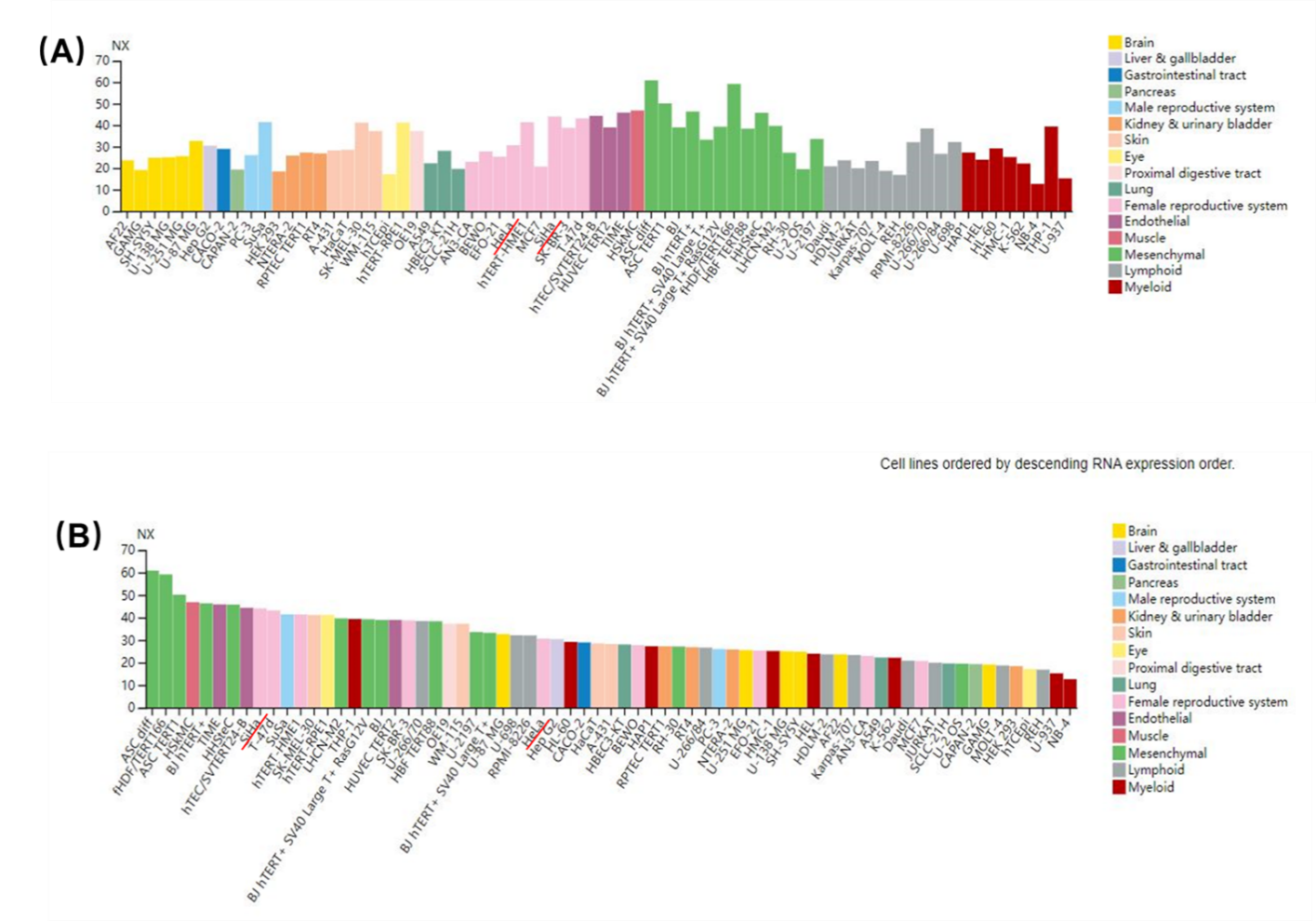
**

Figure S1


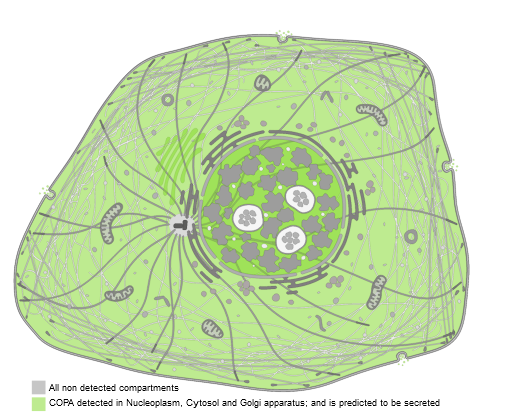


Figure S2


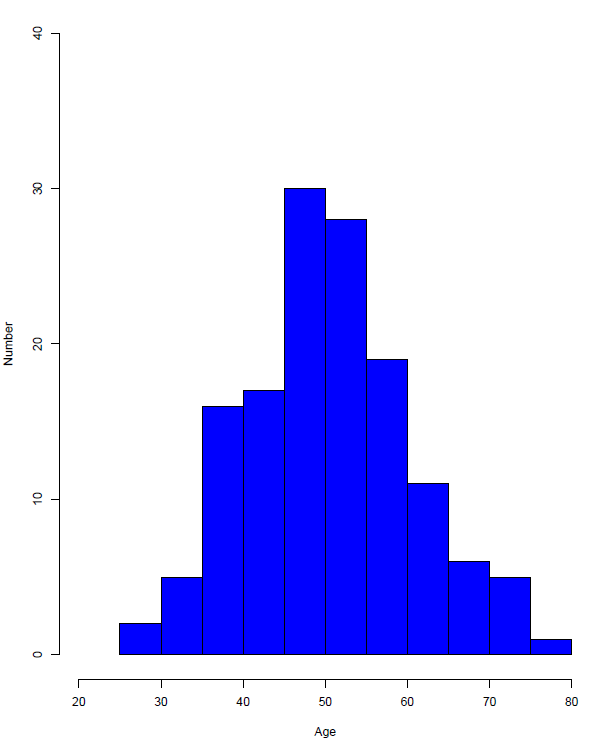


Figure S3


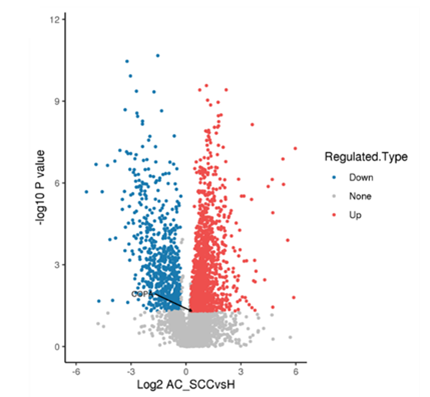


Figure S4
